# Harnessing algae oil as a sustainable DHA source for parenteral nutrition in vegan patients

**DOI:** 10.1038/s41598-025-03319-7

**Published:** 2025-05-27

**Authors:** Joanna Czerniel, Aleksandra Gostyńska-Stawna, Natalia Urbaniak, Karina Sommerfeld-Klatta, Maciej Stawny

**Affiliations:** 1 Department of Pharmaceutical Chemistry, Poznan University of Medical Sciences, 3 Rokietnicka, Poznan, 60-806 Poland; 2 Department of Toxicology, Poznan University of Medical Sciences, 3 Rokietnicka, Poznan, 60-806 Poland

**Keywords:** Omega-3 fatty acids, Intravenous lipid nanoemulsion, Vegan formulation, Clinical nutrition, Drug delivery, Pharmaceutics

## Abstract

Parenteral nutrition (PN) is a life-saving intervention for patients unable to meet their nutritional needs through oral or enteral routes. However, long-term PN therapy is often associated with complications, including intestinal failure-associated liver disease (IFALD), largely attributed, among other factors, to oxidative stress induced by pro-inflammatory unsaturated fatty acids. To mitigate the risk of developing IFALD, NEs have been optimized by increasing the content of Ω-3 fatty acids, particularly docosahexaenoic acid (DHA). This study aimed to develop a novel NE utilizing algae oil as a sustainable source of DHA, along with soybean lecithin as an emulsifier, to create a fully animal-free alternative to commercial intravenous NEs. The formulated algae oil-based NEs met pharmacopeial and physicochemical standards for intravenous administration, achieving a mean droplet diameter below 166.2 nm, a narrow polydispersity index, and a minimal percentage of fat globules larger than 5 μm, capped at a maximum of 0.01%. They demonstrated excellent compatibility with commercial PN admixtures, biocompatibility with red blood cells, and stability over six months of storage. Among the formulations, NE P100, prepared using non-GMO soybean-derived phospholipids containing over 90% phosphatidylcholine, exhibited the most favorable properties, indicating its potential for further development. These findings highlight algae oil as a sustainable and effective source of DHA, offering a viable option for PN-dependent patients, including those following vegan diets, while reducing the risk of IFALD. Further in vitro and in vivo research is warranted to expand applications and refine this vegan alternative in PN therapy.

## Introduction

Currently, many patients rely on parenteral nutrition (PN) in both hospital and outpatient settings. PN is classified as a life-saving procedure and plays a significant role in the treatment of patients ranging from the youngest to the elderly population^1,2^. The administration of parenteral nutrition (PN) has notably improved patient survival rates; however, it is associated with potential adverse effects, including intestinal failure-associated liver disease (IFALD), also known as parenteral nutrition-associated liver disease (PNALD)^1,3^. Depending on the method of administration and the patient’s health condition, the likelihood of developing this disease ranges from 25% to as high as 100%^4^. IFALD leads to steatosis, cholestasis, and ultimately, liver cirrhosis^1^. Its pathomechanism is complex, involving multiple factors, including long-term PN, glucose overloading, phytosterol accumulation, lack of enteral stimulation, catheter-related bloodstream infections, and oxidative stress induced by lipid nanoemulsions (NEs) rich in omega-6 fatty acids^5,6^. The risk of adverse effects associated with long-term PN administration drives contemporary researchers to identify the causes of these phenomena and propose effective preventive strategies. One promising approach involves modifying the composition of PN to increase the level of immunomodulating components, such as Ω-3 fatty acids, while simultaneously reducing the use of soybean oil-based lipid NE^2,7^.

Intralipid, an intravenous lipid NE with a soybean oil-based lipid phase, is a rich source of Ω-6 fatty acids, which, unlike Ω-3 fatty acids, exhibit pro-inflammatory properties that contribute to hepatocyte damage^8^. Both the high soybean oil content and an excessive Ω-6 to Ω-3 ratio are considered probable causes of complications associated with PN administration^9^. Both oral and intravenous supplementation of Ω-3 fatty acids, particularly eicosapentaenoic acid (EPA) and docosahexaenoic acid (DHA), has been associated with hepatoprotection and enhanced liver regeneration^10,11^. Preoperative and subsequent intraoperative intravenous administration of these fatty acids has shown positive effects on the success and safety of liver-related surgeries^12^.

Currently, Omegaven, a commercially available intravenous lipid NE formulated with fish oil as a source of Ω-3 fatty acids, particularly DHA and EPA, is available on the market. Omegaven also contains a higher dose of tocopherol compared to competing NEs^13,14^. This composition supports hepatocyte protection against damage during PN administration^15^. Studies conducted on neonates and young children requiring PN have shown that replacing Intralipid with Omegaven reduced mortality due to irreversible liver damage and led to the resolution of changes characteristic of cholestasis^9^. The mechanism behind Omegaven’s hepatoprotective effect is attributed to its ability to decrease cytokine levels that inhibit the synthesis of paraoxonase 1, an enzyme involved in ester bond hydrolysis and antioxidant processes^14,16^. This results in increased enzyme levels and inhibition of oxidative stress, which otherwise leads to liver cell damage. Omegaven also reduces TGF-β protein levels, the synthesis of which depends on inflammatory factors^14^. Similar to oral administration, intravenous NEs rich in DHA and EPA lower TNF-α levels, an effect not observed with NEs based solely on Ω-6 fatty acids^17^. Reducing inflammation alone contributes to improved liver function^18^. Omegaven is currently introduced as a supplement to complete PN admixtures, providing DHA and EPA as a therapeutic agent for existing liver diseases. It is important to highlight that traditional fish oil, commonly used as a source of DHA in PN, has a highly variable fatty acid composition influenced by factors such as fish families (*Carangidae*,* Clupeidae*,* Engraulidae*,* Osmeridae*,* Salmonidae*, or *Scombridae* *sp*.), diet, and environmental conditions. Pharmacopoeial requirements apply only to the content of Ω-3 fatty acids, which state that the sum of EPA and DHA must be a minimum of 45%, and the total content of Ω-3 fatty acids must not be less than 60%^19^. Complete replacement of the standard NE with Omegaven is not recommended because it does not provide sufficient α-linolenic acid and linoleic acid, potentially leading to deficiencies of certain essential fatty acids. However, clinical studies and animal trials have not confirmed this hypothesis^9^.

The DHA and EPA in Omegaven are sourced from fish oil, whose production is often viewed as unsustainable due to overfishing and its harmful impact on marine ecosystems. However, algae—the primary food source for fish—are the original, natural producers of these omega-3 fatty acids and represent a promising, sustainable alternative. Moreover, the bioactive components of algae show a wide spectrum of biological activity, and algal oil-based formulations can serve as drug delivery systems for hydrophobic compounds^20^. The algae utilize a synthesis method distinct from that of animal cells. Depending on the synthesis conditions, this process follows two pathways: the aerobic pathway, leading to EPA production, and the anaerobic pathway, observed in species like *Schizochytrium sp.*, where DHA and EPA are the main products^21^.

Intravenous lipid NEs also provide phosphatidylcholine (PC) derived from phospholipids (lecithin) used as emulsifiers in these preparations^22^. PC deficiency is considered a contributing factor to hepatocyte dysfunction during long-term PN therapy. The PC content in commercial intravenous NEs varies due to differences in the oil phase composition, particularly the type of oil used^23^. PC sources can be categorized as plant-based or animal-based. Plant-based PC is most commonly derived from soy or sunflower seeds, while animal-derived PC comes from egg yolks^24,25^. All registered intravenous lipid NEs in Europe and the USA use formulations based on egg yolk lecithin^25^. Given PC’s significant role in liver function and its positive effects on lipid and carbohydrate metabolism, increasing its supply, combined with the effects of DHA and EPA delivery, may reduce IFALD risk or enable cholestasis treatment in affected patients.

In summary, to reduce the likelihood of complications associated with PN, it is essential to use PN admixtures with optimal compositions, paying particular attention to the choice of lipid NE. For patients requiring this type of therapy, lipid NE serves as the sole source of fatty acids. Consequently, changes in fatty acid levels during PN administration depend on the type of NE provided. Using intravenous NEs high in Ω-3 fatty acids and low in Ω-6 fatty acids, along with additional DHA and EPA supplementation, can extend survival and reduce complications in patients requiring long-term PN.

The ongoing trend toward healthy lifestyles and environmental protection, including the pursuit of reducing greenhouse gas emissions, directly contributes to the increasing number of people adopting plant-based diets^26^. Unfortunately, no alternative treatment exists for temporary or permanently PN-dependent patients that excludes animal-derived products. With respect for patients’ rights and the growing demand for products that align with diverse dietary preferences, developing a vegan-friendly alternative to existing therapies represents a significant step toward enhancing the acceptance of required treatment^27,28^. Our study aims to develop and evaluate an intravenous NE rich in DHA and EPA that is entirely free of animal-derived products. In addition, we focused on using lecithin with the highest PC content while comparing the type of lecithin used, standardized for PC content, to observe the effect on the physicochemical properties of the NE and its stability.

## Materials and methods

### Materials

The study utilized algae oil derived from *Schizochytrium* sp. (Olini, Poland), alongside Omegaven (Fresenius Kabi, Germany). Soybean-derived phospholipids with 70% PC (Lipoid P 75), non-GMO soybean-derived phospholipids with ≥ 90% PC (Lipoid P 100), egg yolk-derived phospholipids with 80% PC (Lipoid E 80), and sodium oleate (Lipoid Sodium Oleate) were purchased from Lipoid GmbH, Germany. Lipoflex Plus, Lipoflex Special, Lipoflex Peri, Tracutil, and Viantan were obtained from B. Braun Melsungen AG, Germany. All reagents used were of analytical or high-performance liquid chromatography grade.

### Study of algae oil properties

Quality control tests on the algae oil were conducted following the European Pharmacopoeia XII^29^. The investigation began with the characterization of the algae oil to evaluate its suitability for intravenous formulations. Acid value (I_A_) and iodine value (I_I_) were determined using standard methodologies, and results were reported as mean ± standard deviation (SD) from two independent measurements.

### Preparation of intravenous NE

Intravenous NEs were prepared using a high-shear homogenization method followed by high-pressure homogenization. The oil phase, consisting of algae oil and tocopherol, and the aqueous phase, comprising water, glycerol, sodium oleate, and soybean or egg yolk phospholipids, were separately mixed. Three different 10% algae oil-in-water NEs were formulated using soybean-derived phospholipids containing 70% PC, non-GMO soybean-derived phospholipids with over 90% PC, or egg yolk phospholipids with 80% PC.. The qualitative and quantitative composition of the developed NEs is shown in Table 1.

**Table 1:** Qualitative and quantitative composition of the developed NEs.

Ingredient	NE P75	NE P100	NE E80
	[g]		
Algae oil	10.0	10.0	10.0
Lipoid P 75	1.8	–	–
Lipoid P 100	–	1.8	–
Lipoid E 80	–	–	1.8
Tocopherol	0.03	0.03	0.03
Glycerol	2.25	2.25	2.25
Sodium oleate	0.05	0.05	0.05
Water for injection	85.87	85.87	85.87

*Lipoid P 75* soybean-derived phospholipids containing 70% phosphatidylcholine, *Lipoid P 100* non-GMO soybean-derived phospholipids containing ≥ 90% phosphatidylcholine, *Lipoid E 80* egg yolk-derived phospholipids containing 80% phosphatidylcholine.

The aqueous and oil phases were mixed at 65 °C with magnetic stirring at 420 rpm until completely dissolved. The aqueous phase was then gradually combined with the oil phase during mechanical homogenization at 19,000 rpm for 10 min. Subsequently, the obtained core emulsions were subjected to high-pressure homogenization at 800 bar for 10 cycles. Post-homogenization, the pH of the NEs was checked, which was about 7.8, and then the NEs were alkalized with NaOH (as recommended in the literature) to pH ~ 8.5^25^. The NEs thus prepared were sealed under a nitrogen atmosphere and then sterilized at 121 °C for 20 min. After the NEs had cooled completely, the pH was tested again. The method of NEs preparation was developed in our laboratory^30^.

### Physicochemical characterization

The physicochemical properties of the developed NEs (NE P75, NE P100, and NE E80) were evaluated post-sterilization according to the previously published methodology^30,31^. The parameters assessed included mean droplet diameter (MDD), polydispersity index (PDI), percentage of fat residing in globules larger than 5 μm (PFAT5), pH, osmolality (Osm), and zeta potential (ZP). The commercial NE, Omegaven, was used as a reference. MDD and PDI were measured using dynamic light scattering (DLS) with the Zetasizer Nano ZS (Malvern Instruments, United Kingdom), while PFAT5 was analyzed using a light-obscuration/single-particle optical sensing method using the PAMAS SVSS particle counter (PAMAS Partikelmess- und Analysesysteme GmbH, Germany). ZP was determined by the Zetasizer Nano ZS (Malvern Instruments, United Kingdom) and calculated using the Smoluchowski equation based on electrophoretic mobility. pH and Osm were determined using a SevenCompact pH meter (Mettler Toledo, USA) and an Osmometer 800 CLG (Tridentmed, Poland), respectively. All measurements were performed in triplicate.

### Compatibility with PN admixtures

The compatibility studies are based on the methodology by Stawny et al.^32^ and Gostyńska et al.^33^. Three commercial PN admixtures in three-chamber bags (Lipoflex Plus 1250 mL, Lipoflex Special 1250 mL, Lipoflex Peri 1250 mL) were supplemented with 10 mL of trace element solution (Tracutil) and 5 mL of reconstituted vitamin complex (Viantan). The activated and supplemented PN admixtures were combined with the developed NEs or Omegaven in a 12.5:1 ratio, simulating the maximum recommended Ω-3 fatty acid supplementation as specified by the manufacturer of commercial PN admixtures (100 ml of Omegaven per bag). Reference samples to which no supplement NEs were added were also prepared for testing. Physicochemical properties, including MDD, PDI, PFAT5, ZP, pH, and Osm, were measured immediately and after 24 h of storage at 22 ± 2 °C without light exposure.

### Assessment of intravenous administration feasibility

To evaluate the feasibility of intravenous administration, the injectability of developed NEs was tested according to the method developed by Gostyńska et al.^34^. This test was performed using a Perfusor Compact Plus syringe pump (B. Braun, Germany) equipped with a 60 mL syringe and injection needles with sizes ranging from 27 G to 19 G. The evaluation was performed using infusion rates between 25 and 200 mL/h. Pressure below 75 mmHg was required for successful administration. Higher pressures triggered alerts and pump shutdown, which was considered a sign of a lack of injection feasibility.

### Biocompatibility assessment

Hemolysis tests were performed to assess the biocompatibility of the developed formulations and the safety of their intravenous administration. 6 mL of red blood cell concentrate (collected from healthy volunteers with blood type A) was mixed with 80 µL of normal saline (positive control), 80 µL Triton X-100 (negative control), or 80 µL of NEs diluted in 2 mL phosphate-buffered saline (PBS, pH 7.4). Samples were incubated at 37 °C for 1 h, centrifuged at 1200 rpm for 10 min, and supernatants were analyzed for hemoglobin (HGB) concentration using the XN-10 hematology analyzer. The hemolysis degree (%) was calculated using the formula:

$${\text{Hemolysis}} (\%) =\:\:\:\frac{HGB\:\left(positive\:control\right)\:-\:HGB\:\left(sample\right)\:}{HGB\:\left(positive\:control\right)\:-\:HGB\:\left(negative\:control\right)}\times\:100$$

### Stability assessment

The developed NEs were stored under three different storage conditions: 4 ± 1 °C, 22 ± 2 °C, and 30 ± 2 °C with 75 ± 5% humidity, without light exposure. Physicochemical evaluations were performed at predetermined intervals, after 1, 2, 4, and 8 weeks, and after 6 months of storage, following the methodologies outlined in Section “Physicochemical characterization”.

### Statistical analysis

The results are presented as mean values accompanied by SD. Statistical analysis was performed using Statistica 12 software (StatSoft, Krakow, Poland). A one-way analysis of variance (ANOVA) was applied to evaluate the statistical differences between the samples. A significance threshold of *p* < 0.05 was established prior to the analysis.

## Results and discussion

The study aimed to develop intravenous lipid NEs with high PC and Ω-3 fatty acid content based on algae oil. The qualitative and quantitative composition of the studied NEs is presented in Table 1. Such NEs may serve as an alternative source of Ω-3 fatty acids for supplementation in PN admixtures compared to the currently available pharmaceutical product, Omegaven. Adding Ω-3-rich NEs to PN admixtures increases the proportion of EPA and DHA, thereby enhancing their immunomodulatory effects^35^. Currently, Omegaven is the only preparation available specifically designed to supplement PN admixtures with additional Ω-3 fatty acids. Its composition, based on fish oil and egg yolk phospholipids, makes it unsuitable for vegan patients. Furthermore, as awareness of the significance of Ω-3 fatty acids in the diet grows, the demand for such preparations increases. Therefore, identifying alternative sources of EPA and DHA for producing intravenous lipid NEs becomes crucial. Marine algae are a primary source of unsaturated Ω-3 fatty acids, and their cultivation is relatively simple^21^. According to the manufacturer, the algae oil used in this study contains 0.6 g EPA and 45 g DHA per 100 g of oil, which, converted to a 10% NE, gives a theoretical content in 100 g of formulation equal to 0.06 g of EPA and 4.5 g of DHA. In contrast, Omegaven’s composition includes 1.25–2.82 g EPA and 1.44–3.09 g DHA per 100 g of NE^13^. This indicates that using algae oil to develop a 10% lipid NE equivalent to Omegaven may result in preparation with even higher DHA content within the same volume. Due to its low EPA content, algae oil should not be considered a sustainable source of EPA.

Another significant factor influencing the quality of the developed NEs is the type of emulsifier used. Three NEs (NE P75, NE P100, and NE E80) were formulated, differing solely in their emulsifiers: soybean lecithin (Lipoid P 75 and Lipoid P 100) and egg yolk lecithin (Lipoid E 80), respectively. These three emulsifiers were chosen after thoroughly analyzing the literature. It was observed that the egg yolk lecithin most often chosen by researchers was Lipoid E 80. In the case of soybean lecithin, Lipoid S 75 is the most commonly used in research^36^. Lipoid company distinguishes soy lecithin into two subtypes, referred to by the letters "S" (from genetically modified soybeans) and "P" (from non-GMO soybeans). In both cases, the manufacturer defines the same PC content, above 70%, but our choice was P 75 for environmental reasons. Lipoid P 100 also comes from non-GMO cultivation, but the standardized PC content was set at a minimum of 90%^37^. Due to the topic of the research, in which we sought to increase the amount of pure plant-derived PC, we chose the soybean phospholipids with the highest content of PC. On such grounds, Lipoid E 80, P 75, and P 100 were finally selected for the study. Egg yolk lecithin is commonly used as an emulsifier in the industrial production of intravenous lipid NEs. However, due to its lower oxidative stability compared to soybean lecithin, attempts were made to replace egg yolk lecithin with soybean lecithin^36^. The composition of the developed NEs was compared to that of Omegaven, as shown in Table 2. The mass of phospholipids in the developed NEs was increased relative to Omegaven, reaching 1.8 g/100 g of NE in all formulations (Table 2).

**Table 2:** Comparison of the composition of developed NEs and Omegaven.

Sample	Ω-3 source	Oil phase quantity [%]	Emulsifier content [g/100 g of NE]	Emulsifier trade name	Emulsifier composition
NE P75	Algae oil	10	1.8	Lipoid P 75	soybean phospholipids containing 70% PC
NE P100				Lipoid P 100	soybean phospholipids containing ≥ 90% PC
NE E80				Lipoid E 80	egg yolk phospholipids containing 80% PC
Omegaven	Fish oil		1.2	No data	purified egg yolk phospholipids

*PC* phosphatidylcholine.

### Analysis of algae oil properties

Quality control tests on the algae oil were conducted following the European Pharmacopoeia XII^29^. Two key fat characteristics were determined: I_A_ and I_I_. The I_A_ reflects the amount of free fatty acids in the oil, while the I_I_ indicates the number of unsaturated bonds in the molecule. The mean I_A_ obtained in the study was 0.41 ± 0.06, within pharmacopeial requirements, which set a maximum limit of 0.5^29^. The I_I_ of 82.84 ± 0.66 mg confirms the presence of unsaturated bonds, such as those found in EPA and DHA. Comparatively, fish oil derived from *Opisthopterus tardoore* demonstrated the I_I_ of 121.9 ± 2.14 mg, indicating a higher degree of unsaturation in this fish oil compared to the studied algae oil. However, the I_I_ of the fish oil used in the preparation of Omegaven remains unknown, limiting direct comparisons.

### Physicochemical properties of the developed NEs

The physicochemical properties of the NEs were evaluated based on criteria established by the United States Pharmacopeia (USP) for intravenous lipid emulsion^38^. Particle size was evaluated using two methods: DLS to determine the MDD, with an acceptable threshold of < 500 nm, and light obscuration to assess the PFAT5, with an acceptance limit of ≤ 0.05%^38^. It is essential to remember that the average capillary size is ~ 5–10 μm. According to this limitation, the droplet size of NE is crucial in intravenous administration to avoid embolization of the blood vessels^39^. The PDI was also assessed to evaluate the homogeneity of the developed oil-in-water systems, with values ≤ 0.25 deemed acceptable for parenteral applications^40^. Literature data indicate that when ZP approaches zero, oil droplets encapsulated in micelles tend to flocculate, which can destabilize the formulation. The resulting NEs are expected to have a negative ZP of about − 30 mV and lower, which, according to literature data, characterizes stable formulations^41^. ZP at this level was achieved by adding a small amount of the anionic surfactant sodium oleate (0.05% (*w/w*)), also present in Omegaven. The lecithin in the NEs has a protective effect, so the sodium oleate does not exhibit typical hemolytic activity in such a system^42,43^.

Comprehensive physicochemical analysis, including MDD, PDI, ZP, pH, and Osm immediately after preparation and during storage was performed. The results indicated no substantial differences in physicochemical parameters between NEs obtained using different emulsifiers. However, NE P75 and NE E80 exhibited lower ZP values, indicating potentially greater stability than NE P100. Notably, all developed NEs demonstrated MDD values over 30% smaller than those of Omegaven while presenting comparable results for other physiochemical parameters (Table 3).

**Table 3:** Comparison of physicochemical parameters of developed NEs and Omegaven.

Parameter	NE P75	NE P100	NE E80	Omegaven
MDD [nm]	164.0 ± 1.3^	161.1 ± 2.6^	166.2 ± 0.8^	237.8 ± 1.2
PDI	0.11 ± 0.00	0.11 ± 0.01	0.08 ± 0.01	0.10 ± 0.02
PFAT5 [%]	0.002 ± 0.000^	0.001 ± 0.000^	0.010 ± 0.000^	0.000 ± 0.000
ZP [mV]	− 33.3 ± 1.4^	− 24.2 ± 1.6^	− 38.3 ± 0.3^	− 37.3 ± 0.3
pH*	7.9^	7.7^	7.5^	8.0
Osm [mOsm/kg]	270	268	268	344

*Standard deviation value was less than 0.01 for all formulations, ^Result statistically significant compared to the Omegaven (*p* = 0.05), *MDD* mean droplet diameter, *PDI* polydispersity index, *PFAT5* a percentage of fat residing in globules larger than 5 μm, *ZP* zeta potential,* Osm* osmolality.

In conclusion, the physicochemical parameters of the obtained algae oil-based NEs met pharmacopoeial standards and literature limits for intravenous administration and were characterized by significantly lower MDD than Omegaven.

### Compatibility with PN admixtures

**Fig. 1:**
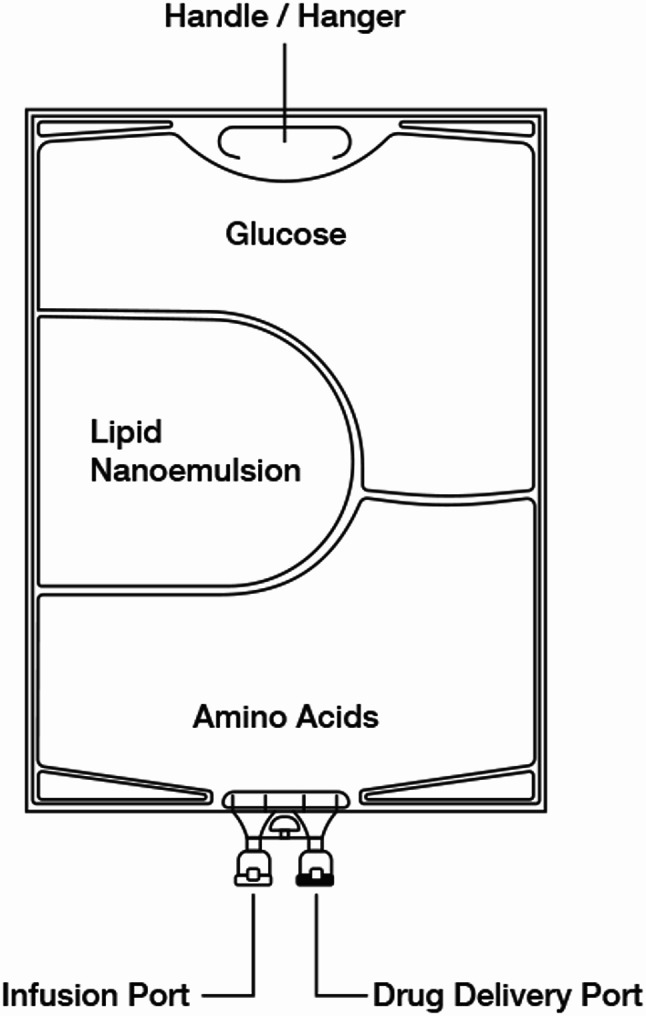
The construction of a three-chambered bag containing parenteral nutrition (PN) admixture.

Patients receiving long-term PN based on first- or second-generation intravenous lipid NEs lacking fish oil may develop an Ω-3 fatty acid deficiency, necessitating supplementation. In such cases, it is recommended to include Omegaven, which is rich in EPA and DHA^9^. If the simultaneous administration of NE with the corresponding PN admixture is required, their compatibility must be thoroughly evaluated. Supplementing PN admixtures with additional components, such as an additional intravenous lipid NE, can result in pharmaceutical phase interactions. Incompatibilities may arise between active substances, excipients, or even packaging materials, potentially leading to destabilization of the oil-in-water system. In the case of intravenous administration, such instability poses a significant risk, including vascular embolization caused by the presence of particles with inappropriate sizes^44^. To evaluate the potential of the NEs developed in this study as an alternative source of Ω-3 fatty acids to Omegaven for supplementing PN admixtures, compatibility tests were performed. Specifically, the developed NEs were added to three commercial PN admixtures that differed in the quantitative composition of amino acids, glucose, fatty acids, and electrolytes. All formulations were supplemented with vitamins and trace elements to ensure a comprehensive assessment (Fig. 1).

Key parameters such as MDD, PDI, PFAT5, ZP, pH, and Osm were analyzed immediately after preparation and following 24 h of storage at 22 ± 2 °C, protected from light. Activated and supplemented with trace elements and vitamins, commercial PN admixtures without the addition of developed NEs were used as reference samples (blanks).

**Fig. 2:**
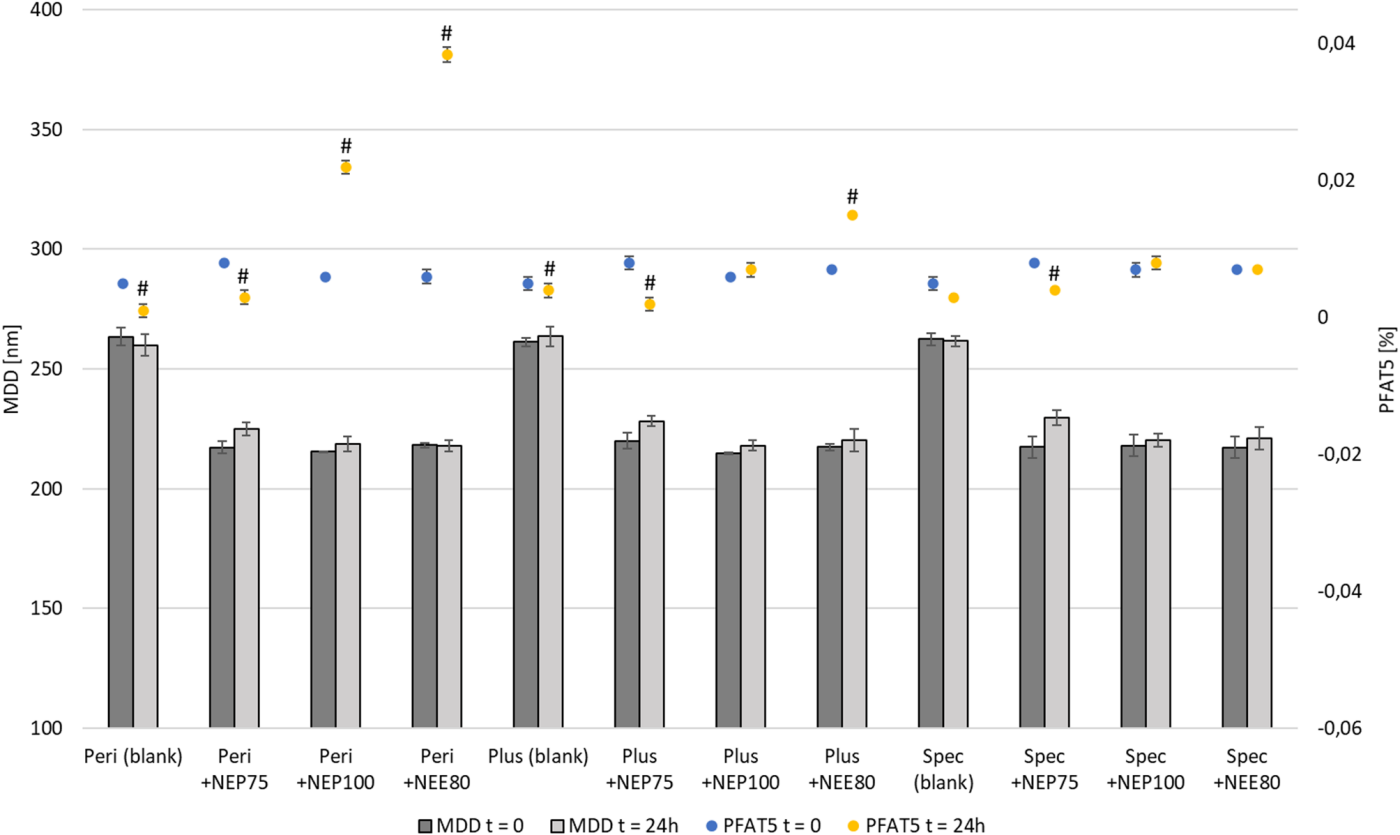
Results of lipid NEs particle size in the compatibility test. *Peri* Lipoflex peri, *Plus* Lipoflex plus, *Spec* Lipoflex special, *MDD* medium droplet diameter, *PFAT5* a percentage of fat residing in globules larger than 5 μm, ^#^Result statistically significant compared to the measurement at time t = 0 (*p* = 0.01).

The compatibility studies revealed that the addition of the developed NEs to commercial PN admixtures resulted in reduced MDD and Osm alongside increased PFAT5 levels (Fig. 2; Table 4). When combined with Peri, there is a noticeable increase in PFAT5 after 24 h to 0.038% and 0.022% for NE E80 and NE P100, respectively (Fig. 2). Nevertheless, like all others, these mixtures do not exceed the USP limit of 0.05%^38^. The other parameters tested (PDI, ZP, and pH) oscillated around similar values (Table 4).

**Table 4:** Results of the compatibility test.

Sample	ZP [mV]		pH*		PDI		Osm [mOsm/kg]	
	t = 0	t = 24 h	t = 0	t = 24 h	t = 0	t = 24 h	t = 0	t = 24 h
Peri (blank)	− 27.4 ± 0.9	− 20.5^#^ ± 0.5	5.3	5.3	0.13 ± 0.01	0.15 ± 0.01	920	921
Peri + NE P75	− 24.8 ± 0.5	− 25.3 ± 0.2	5.3	5.3^#^	0.17 ± 0.01	0.15 ± 0.02	867	872
Peri + NE P100	− 22.7 ± 0.5	− 19.3^#^ ± 0.3	5.3	5.3^#^	0.15 ± 0.02	0.15 ± 0.02	869	873
Peri + NE E80	20.7 ± 0.2	− 25.7^#^ ± 0.7	5.3	5.3^#^	0.13 ± 0.02	0.15 ± 0.02	871	870
Plus (blank)	− 18.7 ± 0.3	− 18.7 ± 0.1	5.3	5.3^#^	0.11 ± 0.03	0.13 ± 0.03	1427	1424
Plus + NE P75	− 22.2 ± 0.7	− 24.7^#^ ± 0.6	5.3	5.3	0.15 ± 0.04	0.16 ± 0.02	1344	1339
Plus + NE P100	− 18.1 ± 0.4	− 17.5 ± 0.4	5.3	5.3	0.18 ± 0.01	0.17 ± 0.01	1335	1345
Plus + NE E80	− 21.6 ± 0.2	− 20.0^#^ ± 0.3	5.3	5.3	0.16 ± 0.01	0.13^#^ ± 0.01	1326	1340
Special (blank)	− 19.9 ± 0.9	− 17.2^#^ ± 0.4	5.4	5.3^#^	0.11 ± 0.03	0.14 ± 0.02	1862	1868
Spec + NE P75	− 20.7 ± 0.2	− 20.8 ± 0.3	5.4	5.4	0.14 ± 0.00	0.14 ± 0.02	1750	1726
Spec + NE P100	− 16.9 ± 0.3	− 17.0 ± 0.6	5.4	5.4	0.15 ± 0.01	0.16 ± 0.01	1739	1747
Spec + NE E80	− 18.1 ± 0.5	− 19.2 ± 0.3	5.4	5.3^#^	0.16 ± 0.02	0.14 ± 0.01	1743	1750

*Standard deviation value was less than 0.01 in all samples. *Peri* Lipoflex peri, *Plus* Lipoflex plus, *Spec* Lipoflex special, *PDI* polydispersity index, *ZP* zeta potential, *Osm* osmolality, ^#^Result statistically significant compared to the measurement at time t = 0 (*p* = 0.01).

Despite these changes, all NEs met pharmacopeial limits, confirming their safety and compatibility with commercial PN admixtures^38^. Accordingly, it was confirmed that the developed NEs could be added to commercial PN admixtures at a rate equivalent to 100 mL per 1250 mL of PN admixture.

### Assessment of intravenous administration feasibility

The obtained algae oil-based NEs provide a promising alternative source of DHA acid for patients who require PN but do not consent to fish oil supplementation. In order to determine the rheological properties of the developed NEs allowing their intravenous administration, an injectability test was performed. For this purpose, according to the method developed by Gostyńska et al.^34^, the possibility of injecting the developed NEs at increasing rates through needles with cross-sectional diameters ranging from 27 to 19 G was determined. The results obtained were compared with the possibility of such administration of the commercial NE Omegaven, which was the reference sample (Table 5).

**Table 5:** Results of injectability test.

Formulation	Infusion rate [mL/h]	Needle diameter [G]						
		27	25	23	22	21	20	19
NE P75	200	−	−	−	−	−	−	+
	100	−	−	−	−	−	+	+
	50	−	+	+	+	+	+	+
	25	+	+	+	+	+	+	+
NE P100	200	−	−	−	−	−	−	+
	100	−	−	−	+	+	+	+
	50	−	+	+	+	+	+	+
	25	+	+	+	+	+	+	+
NE E80	200	−	−	−	−	+	+	+
	100	−	−	−	−	+	+	+
	50	−	+	+	+	+	+	+
	25	+	+	+	+	+	+	+
Omegaven	200	−	−	+	+	+	+	+
	100	−	+	+	+	+	+	+
	50	−	+	+	+	+	+	+
	25	+	+	+	+	+	+	+

“−” infusion not possible (triggering occlusion pressure alarm), “+” infusion possible.

At an infusion rate of 200 ml/h, administering the NE P75 and NE P100 samples was only possible using a needle with the largest diameter of 19 G. In contrast, for the NE E80, administration could be performed with a needle size of 21 G, comparable to the reference NE. The minimum needle diameter through which the tested NEs could flow decreased as the infusion rate decreased. Considering that intravenous injections typically use needles sized 21–23 G, and in cases of administering large fluid volumes, such as in PN, even 20 G needles, the developed lipid NEs were viable for administration at a maximum rate of 100 ml/h for such needle sizes. It is also worth noting that, according to the product characteristics of the Omegaven, the maximum infusion rate should not exceed 0.5 ml/kg body weight/hour^13^. Assuming the NE is administered to patients weighing between 40 and 100 kg, the infusion rate should not exceed 20–50 ml/h. Therefore, using the recommended infusion rates, the administration of the developed lipid NEs would be feasible.

### Biocompatibility assessment

The hemolysis test was conducted to evaluate the safety of intravenous administration of the tested NEs. For this purpose, the hemolytic effects of NE P75, NE P100, and NE E80 were assessed in comparison to positive and negative controls. Additionally, the impact of the reference NE, Omegaven, on erythrocytes was evaluated. The results demonstrated that the developed NEs exhibited the same or lower hemolytic effects on red blood cells as the Omegaven (Table 6). According to the literature, a hemolysis level below 10% is considered acceptable for intravenous formulations^45^. The results obtained in this study indicate that the developed NEs met this criterion and are therefore suitable for intravenous administration.

**Table 6:** Results of hemolysis assay.

Sample	Hemoglobin concentration [g/dL]	Percent of hemolysis [%]
0.9% NaCl	0.0 ± 0.0	n.a.
Triton X-100	14.3 ± 0.2	n.a.
NE P75	0.6 ± 0.1	4.21 ± 0.70
NE P100	0.1^ ± 0.1	0.47 ± 0.40^
NE E80	0.5 ± 0.2	3.27 ± 1.07
Omegaven	0.6 ± 0.1	4.20 ± 0.70

*n.a.* not applicable, ^Result statistically significant compared to the Omegaven (*p* = 0.05).

### Stability studies

**Fig. 3:**
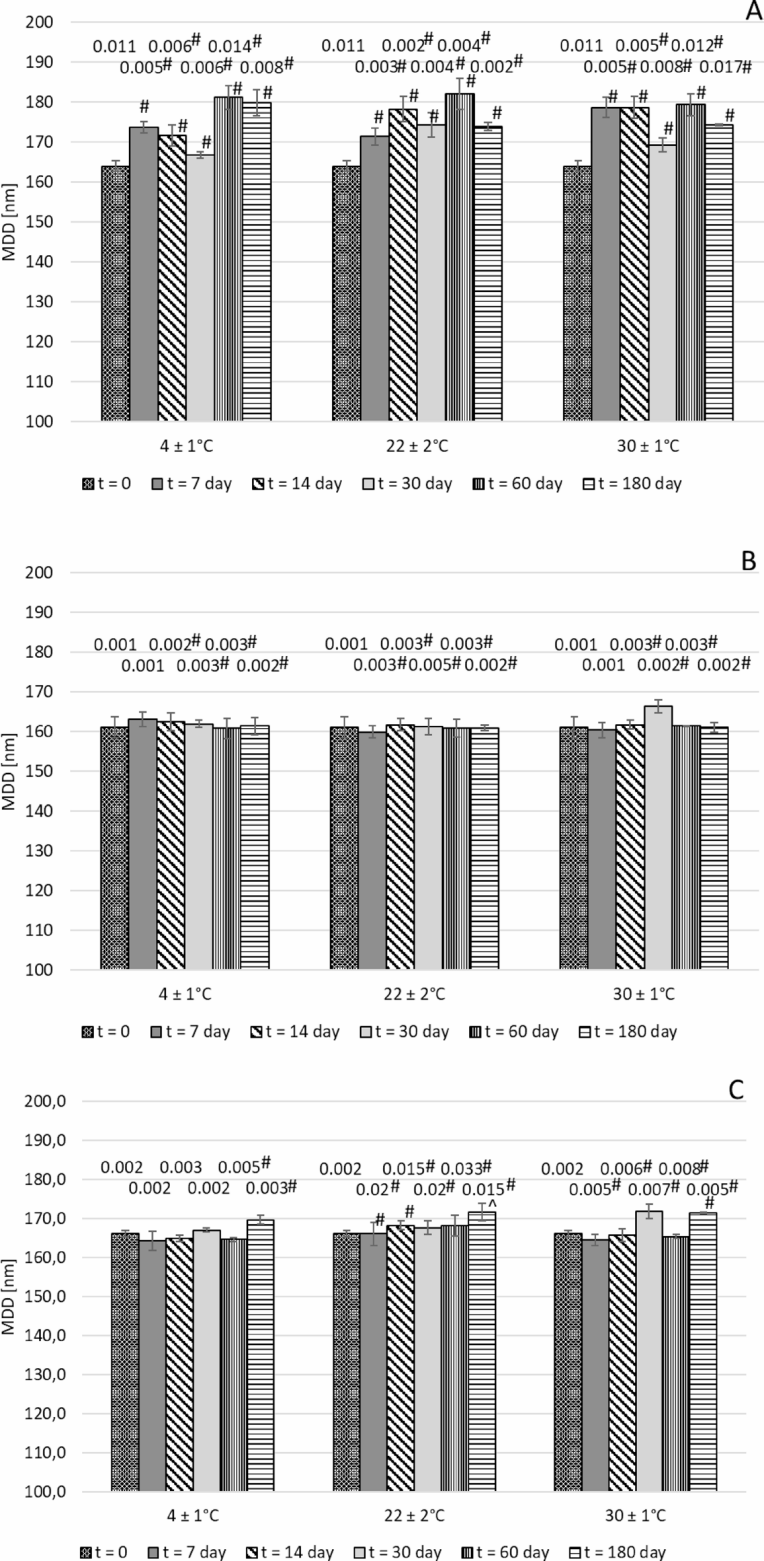
Results of MDD (y-axis) and PFAT5 (values above the bars expressed in %) during the 180-day storage period for NE P75 (**A**), NE P100 (**B**), and NE E80 (**C**), ^#^Result statistically significant compared to the measurement at time t = 0 (*p* = 0.01), ^Result statistically significant compared to the measurement at time t = 0 (*p* = 0.05).

Determining the changes in physicochemical parameters of the NE over time during storage is crucial for assessing its safety for use and establishing its shelf life. Stability studies were performed over six months under three different storage conditions, including refrigerated and ambient storage temperature as well as accelerated aging tests at 30 ± 2 °C and 75% humidity. Critical parameters like MDD, PFAT5, ZP, and pH were monitored over time to evaluate mid-term stability. The results of NEs lipid droplet sizes are presented in Fig. 3.

The largest fluctuations in MDD during the mid-term stability study were observed for NE P75 under all tested conditions. The maximum increase in MDD during the study was 17.2, 18.1, and 15.4 nm for conditions of 4 ± 1 °C, 22 ± 2 °C, and 30 ± 2 °C with 75% humidity, respectively (Fig. 3A). For NE P75 under accelerated aging conditions, a significant increase in PFAT5 was also noted after two months of storage, reaching a maximum of 0.017%. A notable increase in PFAT5 was also observed for NE E80 stored at 22 ± 2 °C, reaching up to 0.033% (Fig. 3C). The highest stability throughout the study in terms of MDD and PFAT5 was demonstrated by NE P100 (Fig. 3B). Despite differences between individual measurement points, none of the developed NEs exceeded the pharmacopeial limits for MDD and PFAT5 across the entire study and under all storage conditions tested^38^.

When analyzing ZP, a marked increase in this parameter was observed after 180 days for NE P75 and NE E80 under all tested conditions (Table 7). The ZP value increased by 19.3% for NE E80 stored at 22 ± 2 °C and up to 64.3% for NE P75 stored at 30 ± 2 °C with 75% humidity relatively to the initial measurement. A ZP increase was also observed for NE P100, though the changes were much smaller, with the highest increases of 8.3% and 5.8% recorded under conditions of 4 ± 1 °C and 22 ± 2 °C, respectively (Table 7).

**Table 7:** Results of stability studies of developed NEs after 180 days of storage.

Storage conditions	Parameters	t = 0 → t = 180 day					
		NE P75		NE P100		NE E80	
4 ± 1 °C	PDI	0.11 ± 0.00 →	0.14 ± 0.00	0.11 ± 0.01 →	0.12 ± 0.01	0.08 ± 0.01 →	0.12 ± 0.01
	ZP [mV]	− 33.3 ± 1.4 →	− 53.4 ± 2.3	− 24.2 ± 1.6 →	− 26.2 ± 1.1	− 38.3 ± 0.3 →	− 45.7 ± 3.7
	pH*	7.8 →	7.0	7.7 →	7.6	7.5 →	7.3
	Osm [mOsm/kg]	270.0 →	270.5	265.0 →	264.0	268.0 →	267.5
22 ± 2 °C	PDI	0.11 ± 0.00 →	0.13 ± 0.01	0.11 ± 0.01→	0.10 ± 0.02	0.08 ± 0.01 →	0.12 ± 0.01
	ZP [mV]	− 33.3 ± 1.4 →	− 51.1 ± 0.1	− 24.2 ± 1.6 →	− 25.6 ± 0.4	− 38.3 ± 0.3 →	− 48.8 ± 1.7
	pH*	7.8 →	4.8	7.7 →	6.4	7.5 →	6.4
	Osm [mOsm/kg]	270.0 →	276.5	265.0 →	262.5	268.0 →	267.0
30 ± 2 °C, 75% humidity	PDI	0.11 ± 0.00 →	0.14 ± 0.01	0.11 ± 0.01 →	0.12 ± 0.01	0.08 ± 0.01 →	0.11 ± 0.00
	ZP [mV]	− 33.3 ± 1.4 →	− 54.7 ± 5.4	− 24.2 ± 1.6 →	− 30.6 ± 2.0	− 38.3 ± 0.3 →	− 48.3 ± 0.5
	pH*	7.8 →	6.2	7.7 →	7.2	7.5 →	6.8
	Osm [mOsm/kg]	270.0 →	276.0	265.0 →	265.0	268.0 →	267.5

*PDI* polydispersity index, *ZP* zeta potential, *Osm* osmolality.

The increase in ZP absolute value is associated with the release of free fatty acids from both lecithin and oil during the storage of NEs^46^. A rise in the absolute value of the surface charge on the droplet enhances droplet repulsion, thereby stabilizing the NE system. However, excessively high concentrations of free fatty acids in intravenous products pose a potential risk of systemic toxicity^47^. In this case, the significant increase in the absolute value of ZP serves as an indicator of the onset of lecithin destabilization and the associated risk of using the NEs for intravenous administration. The release of free fatty acids is further confirmed by a notable drop in pH to visibly lower values for NE P75 and NE E80 (Table 7).

Mid-term stability assessments over six months under varied storage conditions showed that NE P100 had the most stable properties. Both NE P75 and NE E80 exhibited greater variations in parameters such as MDD, PFAT5, and ZP, suggesting a higher tendency to degradation processes. The obtained results and scientific reports highlight NE P100 as the most promising candidate for further tests and clinical applications due to its superior stability and physicochemical properties in developed NEs. Given that each of the developed NEs is based on the same amount of algae oil, the difference in the amount of free fatty acids released over time under different storage conditions is due to the type of lecithin used. Analysis of the growth of ZP over time showed that Lipoid P 100 proved to be the least susceptible to breakdown in the developed formulations. Other studies confirm reports that soybean lecithin has better oxidative stability and is less prone to hydrolysis than egg yolk lecithin^48,49^. It should be remembered that the composition of naturally derived lecithin is heterogeneous, as it can be viewed as a mixture of acetone-insoluble phosphatides, which consist chiefly of PC, phosphatidylethanolamine, phosphatidylserine, and phosphatidylinositol, combined with various amounts of other substances such as triglycerides, fatty acids, and carbohydrates, which generates differences in physicochemical properties^50^. Otto et al. pointed out the inaccurate and misleading HLB value for lecithins found in the literature and tested some phospholipids to accurately determine each HLB, including Lipoid P75, P100, and E80. Authors revealed that the emulsifiers used in our study exhibited distinct individual phospholipid compositions and HLB values. P100 was characterized by the highest PC content, while simultaneously displaying the lowest levels of phosphatidylethanolamine and lysophosphatidylcholine, which could affect the overall stability of developed NE^51^.

## Limitations

Our study aimed to develop and evaluate an intravenous NE rich in DHA that is entirely free of animal-derived products. Conducting in vivo studies is a necessary step to confirm the efficacy and safety of using NE P100 in patients requiring PN therapy. PN admixtures are among the most complex drugs in modern medicine and pharmacy. Therefore, the preliminary studies conducted are insufficient to confirm the safety of intravenous administration in humans. Any new component introduced into the PN admixture is a potential source of interactions and destabilization of the formulation. The algae oil used does not have intravenous administration indications and, to the best of our knowledge, no studies have been conducted in this direction. This raises concerns that should be addressed before proceeding with in vivo studies. Algae oil should undergo purity testing and assessment for the potential presence of substances that could irritate blood vessel walls. Unlike fish oil, algal oil contains phytosterols, which may influence the IFALD development. At this stage of the research, it was not our goal to study the phytosterol content, but given the promising results of the developed NEs, this topic should undoubtedly be addressed. This will allow us to see whether, despite the relatively higher DHA content of the obtained NEs, the presence of phytosterols will impact the hepatoprotective effect.

## Conclusions

In summary, the use of algae oil as an alternative source of DHA facilitated the development of intravenous lipid NEs that meet pharmacopeial and literature-defined standards. Among the tested emulsifiers, the NE P100 formulated with non-GMO soybean-derived phospholipids containing over 90% PC (Lipoid P100) demonstrated the most favorable physicochemical properties for intravenous administration. The formulation’s suitability for intravenous injection at an infusion rate of 50 mL/h, consistent with PN admixture administration, was also confirmed. Compatibility and biocompatibility tests verified the feasibility of incorporating these NEs into commercial PN admixtures and confirmed their safety for intravenous administration. Furthermore, mid-term stability assessments supported the potential for six-month storage of developed formulations, with NE P100 exhibiting the highest stability. This study highlights NE P100, a NE formulated with algae oil and non-GMO soybean-derived phospholipids, as a sustainable and effective alternative source of DHA. This formulation presents a promising option for patients who cannot or prefer not to consume animal-derived products. Further in vitro and in vivo studies on vegan-friendly NEs, particularly NE P100, are recommended to broaden their therapeutic applications in PN admixtures and advance PN therapy.

## Data Availability

The data generated and analyzed during this study are available from the corresponding author upon reasonable request. All relevant datasets supporting the findings of this study are included in the manuscript.

## Abbreviations

DHA: Docosahexaenoic acid
DLS: Dynamic light scattering
EPA: Eicosapentaenoic acid
I_A_: Acid value
IFALD: Intestinal failure–associated liver disease
I_I_: Iodine value
MDD: Mean droplet diameter
NE: Nanoemulsion
Osm: Osmolality
PC: Phosphatidylcholine
PDI: Polydispersity index
Ph. Eur.: European pharmacopeia
PN: Parenteral nutrition
PNALD: Parenteral nutrition–associated liver disease
PFAT5: Percentage of fat residing in globules larger than 5 μm
SD: Standard deviation
USP: United States Pharmacopeia
ZP: Zeta potential
